# Validity of the scan of postgraduate educational environment domains (SPEED) questionnaire in a rural general practice training setting

**DOI:** 10.1186/s12909-019-1455-8

**Published:** 2019-01-17

**Authors:** Bunmi S. Malau-Aduli, Faith Alele, Carlos Fernando Collares, Carole Reeve, Cees Van der Vleuten, Marcy Holdsworth, Paula Heggarty, Peta-Ann Teague

**Affiliations:** 1 College of Medicine and Dentistry, James Cook University, QLD, Townsville, Australia; 2 School of Health Professions Education, Maastricht University, Maastricht, Netherlands; 3 European Board of Medical Assessors, Maastricht, Netherlands

**Keywords:** Quality of educational environment, GP registrars, Rural postgraduate GP training

## Abstract

**Background:**

The educational environment is critical to learning and is determined by social interactions. Trainee satisfaction translates to career commitment, retention and a positive professional attitude as well as being an important factor in assessing the impact of the training program. This study aimed to validate the Scan of Postgraduate Educational Environment Domain (SPEED) tool and assess its appropriateness in evaluating the quality of General Practice (GP) rural postgraduate educational environment.

**Methods:**

A questionnaire containing the 15-item SPEED tool was administered to GP registrars to examine their perceptions of the educational environment. Principal component analysis (PCA) and exploratory factor analysis (EFA) were used to gather evidences of the validity of the instrument based on its internal structure. Additional validity evidence and reliability estimates were obtained using many-facet Rasch model analysis (MFRM).

**Results:**

The survey was completed by 351 registrars with a response rate of 60%. Parallel analysis performed using principal component analysis and exploratory factor analysis suggests that the SPEED tool is unidimensional. The MFRM analysis demonstrated an excellent degree of *infit* and *outfit* for items and training sites, but not for persons. The MFRM analysis also estimated high reliability levels for items (0.98), training sites (0.95) and persons within training sites (ranging from 0.87 to 0.93 in each training sites). Overall, the registrars agreed that the educational environment had high quality, with most (13 out of 15) of the items rated above 4 out of 5.

**Conclusions:**

This study demonstrated a high degree of validity and reliability of the SPEED tool for the measurement of the quality of the educational environment in a rural postgraduate GP training context. However, when applied in a new setting, the tool may not function as a multidimensional tool consistent with its theoretical grounding.

## Background

The educational environment refers to the physical, emotional and intellectual context in which learning occurs and the perspective of the learner is most commonly used to construct and interpret the quality of the educational environment [1]. The context in which learning occurs may affect the engagement of the learner, their motivation and their perception of the relevance of that learning to themselves [2].

The educational environment has substantial, real and influential effects on the trainee [3] and makes a substantive contribution to the trainee’s success, achievement and satisfaction [4]. Evidence in the literature suggest that trainee satisfaction translates to career commitment, retention and positive professional attitude [5]. Trainee satisfaction has also been highlighted as an outcome measure in evaluating the impact of faculty performance on learner’s training experience [6, 7]. Poor learning and training environments may result in poor safety and poor quality in patient care. The educational environment is key to a doctor’s professional development and should be as much of a focus in adult learning as other elements of teaching such as sharing knowledge and expertise [2]. This is because the educational environment is a social environment which is dynamically impacted by human actions and the interaction with the physical components. The learner forms part of the environment and therefore their presence will influence it.

In the postgraduate educational context, junior doctors are employed by health services, and concurrently learn, whilst providing patient care. Evaluating postgraduate medical trainees’ satisfaction will facilitate the development of effective educational experiences [8]. This is because the learners’ perception of the environment impacts their behaviour and determines the efficacy of the environment for learning [9]. Therefore, perceptions of learners represent a consequential and meaningful measure of the educational environment. The importance of a positive educational environment in medical education has received growing acknowledgement and has stimulated the development of several instruments to assess the quality of the postgraduate educational environment [10–13].

Schonrock-Adema et al. [14] recently developed an instrument (The Scan of Postgraduate Educational Environment Domain - SPEED) that is used to assess the quality of the Post Graduate Medical Education (PGME) environment. SPEED is concise and based on a theoretical framework that emphasises three human environment domains in the medical education context - goal orientation, relationships and organisation [15]. Goal orientation refers to the content of the training program, relationships pertain to the atmosphere and interpersonal relationships within the educational environment; and organisation covers the structure of the program [14, 15]. This instrument was deemed appropriate for evaluation of the educational environment in our unique distributed model of regional/rural/remote postgraduate training setting. Exposure to good learning opportunities promotes rural/remote medical careers [16]. However, to ensure adequacy of clinical supervision in such settings, medical educators need to continually monitor and evaluate the quality of the educational environment [17]. To our knowledge, the validity evidence of the use of the SPEED tool based on its internal structure for the assessment of the educational environment in a rural Generalist Medical Training setting has not been obtained yet. Therefore, this study aimed to validate the Scan of Postgraduate Educational Environment Domain (SPEED) tool and assess its appropriateness in evaluating the quality of General Practice (GP) postgraduate educational training in a rural setting.

## Methods

### Study context

James Cook University (JCU) developed a fully integrated regional training pipeline by establishing a General Practice (GP) training program, formerly known as Generalist Medical Training – GMT in 2016 [18] as an extension to the undergraduate medical program. The program adopts a distributed model to the delivery of general practice training to set a new way forward in growing the rural medical workforce and to assure continuity of care, experience and relationships. The JCU GP educational model is registrar focused, utilising best practice and emerging educational methods and technologies to maximise the learning experience, reduce isolation, and build a future general practice workforce. The model is vertically and horizontally integrated to build communities of practice and teaching and learning organisations within general practice in the region. The program utilises the stated elements of the Royal Australian College of General Practitioners (RACGP) and Australian College of Rural and Remote Medicine (ACRRM) curricula integrated with the distinct features of general practice in this region. In addition to the core curriculum and college standards, these integrated elements include context of practice, population health, Aboriginal and Torres Strait Islander Health, remote technologies and advanced/specialised skills. The JCU GP educational model is a three to four-year full time training program depending on the choice of fellowship (RACGP or ACCRM).

A survey was administered to JCU’s GP registrars to examine their perceptions of the training experience and their level of satisfaction with the educational environment. All enrolled registrars in 2016 and 2017 were invited via email to complete the online survey, which included the 15-items in the SPEED tool, four additional close-ended questions to explore registrars’ overall satisfaction and two open-ended questions to examine their perceptions of the strengths and weaknesses of the educational environment. Participation in the study was voluntary and information on age, gender, location of undergraduate university training (regional, major city or international), and stage of training were included.

The original authors used a 4-point scale ranging from 0 (completely disagree) to 3 (completely agree). They also identified three factors which measured perceptions of the atmosphere, content and organisation of the educational environment. However, in our study, for each of the 15 items in the SPEED tool, respondents were asked to score their agreement to each item (presented as a statement) on a 5-point Likert scale ranging from 1 (completely disagree) to 5 (completely agree). A 5-point scale was used in our study to allow for the inclusion of a midway response (neither agree nor disagree). This was done to enable respondents who were ignorant about or indifferent to a statement to select no opinion instead of being forced to choose a response that did not reflect their true perceptions [19]. A planning panel was set up to review the questions to ensure that they suited our setting and training model. We substituted one question which was not applicable to our setting “Supervisors, nursing staff, other allied health professionals and residents work together as a team here” with “I feel part of the team working here”. The original question was not applicable to the training sites because some of the practices where the registrars are trained are in General practitioner practices in rural areas and do not have the type of organisational structure that the hospitals have. Hence, we modified the question to suit the training environment. Four additional close-ended questions were included to explore registrars’ overall satisfaction with the educational environment. These questions were: “I am satisfied with the facilities offered at this training post”, “The educational resources available at this training post are satisfactory”, “I see a good range of patients and presentations in this training post, I see an appropriate number of patients for my level of training each day” (see Table 1 for an overview of the SPEED items).

**Table 1:** Comparison of loadings (λ) and communalities (*h*^2^) of the items of the SPEED tool obtained in principal component analysis and exploratory factor analysis

Items		PCA		EFA	
		λ	*h* ^2^	λ	*h* ^2^
Item 1	The supervisors are respectful towards registrars	0.82	0.67	0.84	0.70
Item 2	The supervisors are approachable and helpful	0.83	0.69	0.85	0.72
Item 3	I feel part of the team working here	0.79	0.62	0.82	0.68
Item 4	My supervisors are all in their own way positive role models	0.85	0.73	0.87	0.76
Item 5	The training in this post prepares me for my future career	0.78	0.60	0.81	0.65
Item 6	My supervisor supports me in difficult situations e.g. critical incidents	0.84	0.71	0.85	0.73
Item 7	The practice/post staff are clear about my duties and responsibilities	0.81	0.65	0.83	0.68
Item 8	The level of autonomy given to me is appropriate to my level of training	0.82	0.67	0.84	0.70
Item 9	Good clinical supervision is available at all times	0.83	0.68	0.86	0.74
Item 10	There are no staff who have a negative impact on the educational environment	0.63	0.40	0.70	0.49
Item 11	In this placement, ECT visits and / or supervisor reports are useful discussions about my performance	0.59	0.35	0.61	0.37
Item 12	My supervisor prevents me from having to perform too many tasks irrelevant to my learning	0.68	0.46	0.70	0.9
Item 13	My supervisor reserves time to supervise/counsel me	0.72	0.52	0.75	0.56
Item 14	Teaching and learning are emphasised in this post	0.82	0.68	0.83	0.70
Item 15	The feedback provided by my supervisor is focused on my strengths and weaknesses	0.80	0.65	0.81	0.65

### Statistical analyses

#### Validity based on internal structure

Exploratory factor analysis (EFA) and principal component analysis (PCA) were used to determine the extent to which the observed variables can be explained by a smaller number of factors or components, with an assumption of multivariate normality and sample size > 200 [20]. Model evaluation involved inferential tests for factor loadings and goodness of model-fit indices [20]. This parallel analysis was performed under the Kaiser criterion of eigenvalues > 1 for determining the meaningfulness of factors or components. Items are expected to have minimum loadings of 0.3. The suitability of the data for detecting structure was measured using Kaiser-Meyer-Olkin (KMO), with values of 0.6 and above indicating suitability [21]. Bartlett’s test of sphericity at *p* < 0.05 was used to detect if the variables (items) were suitable for structure detection [22]. Results from principal component analysis and exploratory factor analysis from the polychoric correlation matrix were also used to create a scree plot that compares the eigenvalues obtained using the original data with the results from parallel analyses using resampled data with the same size in rows and columns of the original dataset. Goodness-of-fit indices were root mean square error of approximation (RMSEA), and the Tucker-Lewis index (TLI). RMSEA values were considered adequate if below 0.08 and good if below 0.06 [23]. TLI values were considered acceptable if above 0.95 and good if above 0.97 [23]. These analyses were performed using the R package *psych* [24].

#### Reliability

Many-facet Rasch modelling (MFRM) analysis was used to estimate the reliability of each facet of measurement (training sites, persons and items). Rasch modelling uses a series of logarithmic iterations to express items’ difficulties and persons’ abilities in the same latent scale. MFRM is an extension to the original Rasch model that enables the inclusion of other facets of measurement in the latent scale [25]. The facets were considered to have acceptable, good or excellent levels of reliability if the reliability estimate was 0.7–0.8, 0.8–0.9 or > 0.9 respectively [16]. Fit measures, namely *infit* and *outfit*, were also determined for each one of the participating persons, training sites and items. *Infit* refers to inlier-sensitive fit and infit statistics are more sensitive to discrepancies in patterns of responses to items targeting the persons’ ability. In contrast, *outfit* refers to outlier-sensitive fit and *outfit* statistics are more sensitive to items that respondents find relatively very easy or very hard [26]. Values below 2.0 indicate acceptable fit [26]. Group anchoring was applied as it is recommended for unbalanced nested designs, such as the one applied in this study. MFRM analysis were performed using the software FACETS 3.80 [27].

#### Validity based on the relation to other variables

The correlation between participants’ SPEED score and their overall satisfaction score was assessed using a Pearson’s correlation coefficient. Participants’ SPEED score was taken as the percent average of scores for all items, while overall satisfaction score was taken as the percent average scores for the four additional questions. T-test and Analysis of variance (ANOVA) were conducted to determine effects of age, gender, level of training and location of university attended (rural, regional, major city or international) on participants’ responses to the SPEED. The level of significance was set at *p* < 0.05.

### Ethics approval

Ethics approval for the study was obtained from the JCU Human Research Ethics Committee (H6771). Written consent was obtained from study participants.

## Results

Three hundred and fifty-one (351) registrars responded to the feedback survey over the two-year period. Response rate was 60% of the entire cohort of registrars in the JCU GP training program. The mean age of the respondents was 33.82 ± 5.74 years with approximately 55% of the registrars being between 30 and 39 years old. Of the 351 respondents, more than 60% were females; 19% were graduates of universities located in rural regions of Australia, 64% were graduates of universities in major cities, while 15.4% were graduates from international universities.

### Validity based on internal structure

**Fig. 1:**
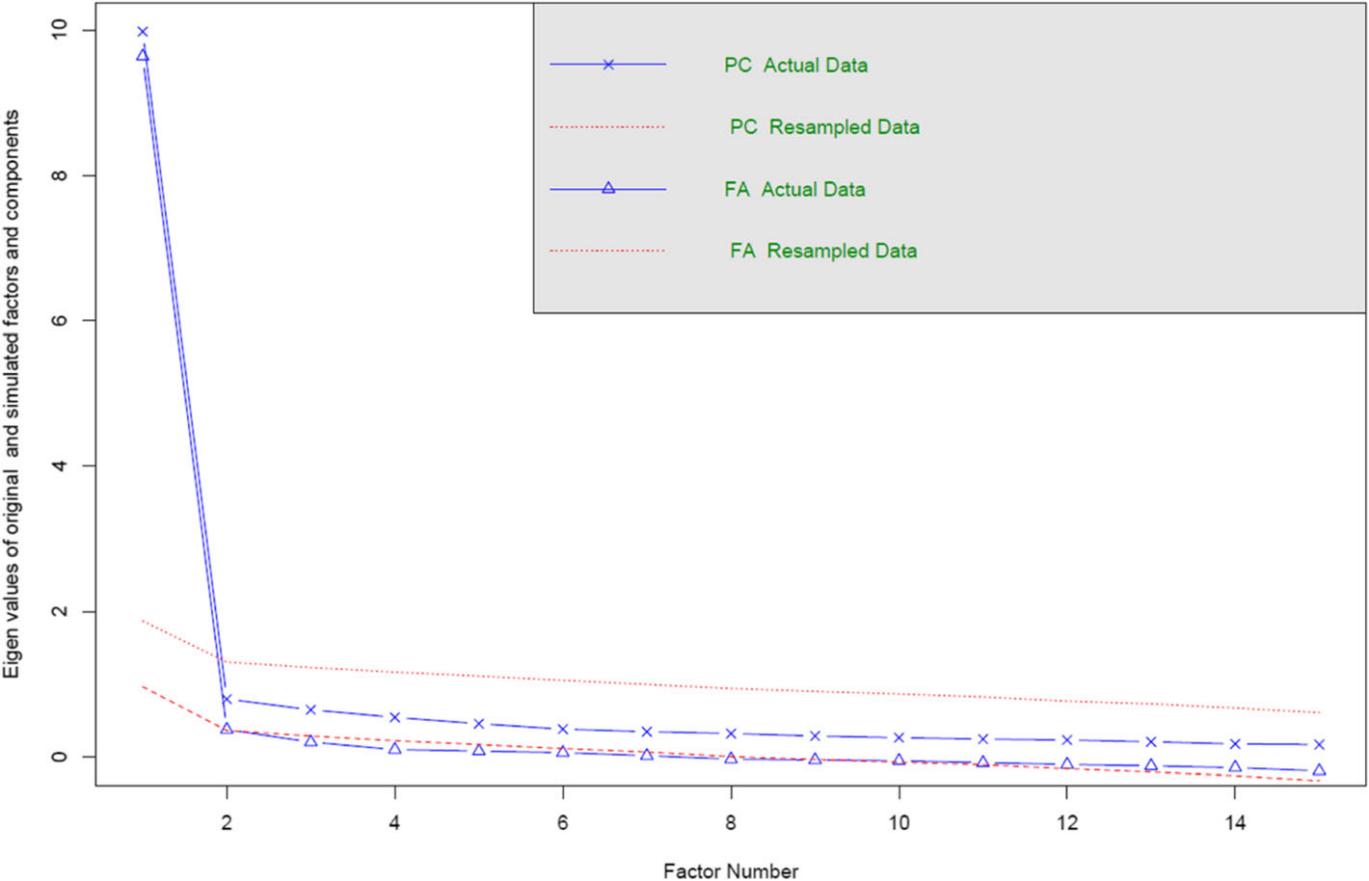
A scree plot of the eigenvalues for the data - (Footnote: Eigen values of tetrachoric/polychoric matrix)

Inspection of the correlation matrix of item responses revealed that all correlation coefficients were above 0.30. The Kaiser-Meyer-Olkin value was 0.959, and Bartlett’s Test of Sphericity was statistically significant (× ^2^ (105) = 3935.367, *p* < 0.001), supporting the suitability of the sample size and the dataset for factor analysis. Measures of sampling adequacy were above 0.943 for all items of the SPEED tool. Principal component analysis resulted in one component with an eigenvalue of 10.717, accounting for 71.45% of the variance; in the exploratory factor analysis, the eigenvalue of the first factor was 10.435, accounting for 60.57% of the variance. All other components had eigenvalues below 1. In comparison with principal component analysis, the exploratory factor analysis also yielded one single factor, with a slightly lower eigenvalue for the first factor (9.53), explaining 63.56% of the variance. Inspection of the scree plot showed a clear distinction between the first component and the second component (Fig. 1).

EFA also produced acceptable goodness-of-fit indices (Tucker-Lewis Index (TLI) = 0.934, root mean square error of approximation (RMSEA) = 0.092). Loadings and communalities for the principal component analysis and the exploratory factor analysis are shown in Table 1.

### Many-facet Rasch model analysis

**Fig. 2:**
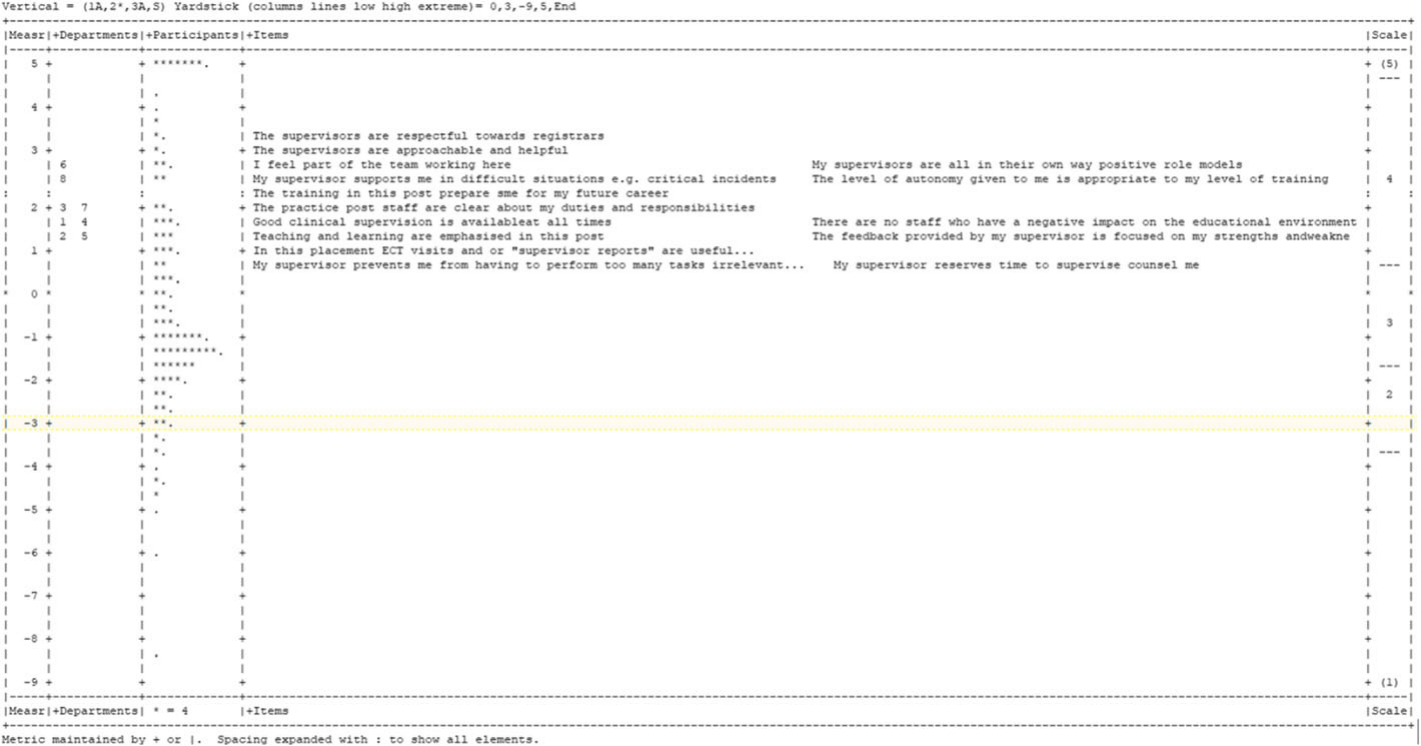
Wright variable map displaying relationships between the distributions of the facet elements on the latent scale. Each asterisk represents 4 persons

The Wright map (shown in Fig. 2) from the many-facet Rasch model (MFRM) enabled an overall visualisation of all facets of measurement on the latent scale. The items which received highest scores were “*The supervisors are respectful towards registrars*” and “*The supervisors are approachable and helpful*”. The lowest scoring items were “My supervisors prevents me from having to perform too many tasks irrelevant to my learning” and “My supervisor reserves time to supervise counsel me”. The last two items are closely located to the boundary location where the Likert scale categories 3 and 4 meet. In the Wright map, it is also possible to observe that the training sites, the object of measurement, have a considerably large distribution, spread through almost 2 logits (standard deviation = 0.41 logits). This is desirable because, analogously to generalisability theory, a large variance for the object of measurement is essential for the overall reliability of the tool. The distribution of items is larger, with a standard deviation of 0.85 logits in this sample.

#### Validity

Fit indices were excellent for training sites and items, with all *infit* and *outfit* mean square measures below 1.5. However, roughly 10% of the participants had *infit* and *outfit* mean square measures above 2.0, which suggests that these participants may have been inattentive while answering the questionnaire. From the 5160 observations, only 54 were unexpected responses with poor fit to the model, showing standardized residuals above 3 (1,05%). The fit results can be considered validity evidence based on internal structure. Category statistics, in which the Rasch-Andrich and Rasch-Thurstone thresholds of the Likert scale followed an ascending order for all categories, can also be considered a positive evidence of validity based on internal structure.

#### Reliability

Reliability for training sites was 0.95 for the sample, with a RMSE (root mean square error) = 0.10 and a separation = 4.19. This means it is possible to reliably classify the training sites into 4 different levels. Reliability for items was 0.98, with a RMSE = 0.12. Reliability for persons was calculated separately for each training sites, due to the group anchoring procedure, necessary to deal with the unbalanced nested design. Reliability for persons within training sites ranged from 0.87 to 0.93. The RMSE for persons within training sites ranged from 0.62 to 0.83, reflecting the poor fit observed for the participants. All reliability estimates refer to the sample, not the population, and include extreme cases.

### Validity based on the relation with other variables

**Fig. 3:**
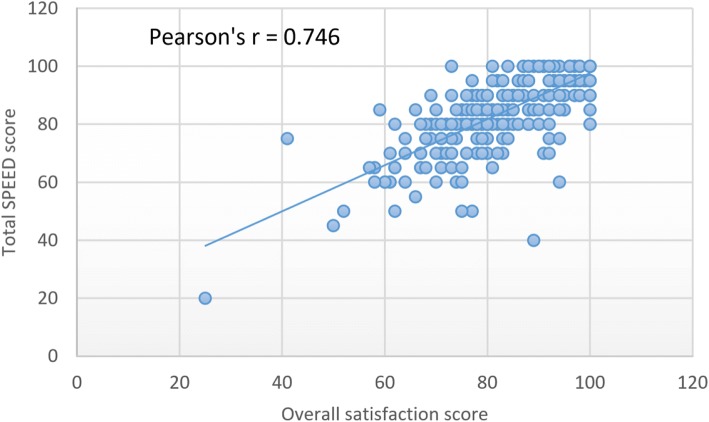
Correlation between participants’ total SPEED and overall satisfaction scores

Participants’ overall satisfaction score was 92.3%. There was a positive linear correlation between the overall SPEED score and the overall satisfaction score (*r* = 0.746; *p* < 0.001; Fig. 3). Mean scores and item-to total score correlations for each of the 15-items are presented in Table 2. The items with the least mean scores were “My supervisor prevents me from having to perform too many tasks irrelevant to my learning and my supervisor reserves time to supervise/counsel me” (3.92 ± 0.77) and 3.98 ± 0.92 respectively). The item “The supervisors are respectful towards registrars” had the highest mean score (4.52 ± 0.61). Of all the demographic variables considered, only gender had a significant influence on the satisfaction with the educational environment. Female registrars gave significantly higher (*p* = 0.02) satisfaction ratings than their male counterparts for two of the SPEED items – “The practice/post staff are clear about my duties and responsibilities” and “My supervisor reserves time to supervise/counsel me”.

**Table 2:** Items in the SPEED with their adjusted item to correlation coefficients

Items		Mean	SD	Item-to-total correlation
Item 1	The supervisors are respectful towards registrars	4.57	0.56	0.75
Item 2	The supervisors are approachable and helpful	4.51	0.61	0.76
Item 3	I feel part of the team working here	4.46	0.68	0.68
Item 4	My supervisors are all in their own way positive role models	4.44	0.66	0.77
Item 5	The training in this post prepares me for my future career	4.37	0.70	0.71
Item 6	My supervisor supports me in difficult situations e.g. critical incidents	4.35	0.68	0.83
Item 7	The practice/post staff are clear about my duties and responsibilities	4.29	0.67	0.75
Item 8	The level of autonomy given to me is appropriate to my level of training	4.35	0.62	0.74
Item 9	Good clinical supervision is available at all times	4.27	0.77	0.76
Item 10	There are no staff who have a negative impact on the educational environment	4.22	0.79	0.65
Item 11	In this placement, ECT visits and / or supervisor reports are useful discussions about my performance	4.05	0.72	0.50
Item 12	My supervisor prevents me from having to perform too many tasks irrelevant to my learning	3.99	0.78	0.60
Item 13	My supervisor reserves time to supervise/counsel me	4.04	0.95	0.71
Item 14	Teaching and learning are emphasised in this post	4.17	0.79	0.80
Item 15	The feedback provided by my supervisor is focused on my strengths and weaknesses	4.18	0.70	0.75

Thematic analysis of the open-text comments from the registrars revealed their perceptions in relation to the strengths of the educational environment and possible areas for improvement. Illustrative quotes are presented in Table 3.

**Table 3:** Sample Comments from Registrars

Theme	Illustrative Quotes
*Strengths of the educational environment*	
*Supportive and accessible supervisors*	*“Very approachable and supportive supervisor who has excellent knowledge and approach to general practice and keen dedication to teaching” (Respondent 333, Male, GPT1/PRT1)*
	*“Excellent support, good supervisor role modelling, strong clinical knowledge of supervisor” (Respondent 112, Male, GPT2/PRT2)*
	*“Excellent supervision, good case load, variety of cases, good opportunities for learning and outreach work” (Respondent 117, Female, Extended Skills)*
*Good Teamwork*	*“Extremely supportive environment for registrars and training: positive workplace and fantastic teamwork; easily accessible supervisor” (Respondent 138, Female, GPT3/PRT3)*
	*“Great supervisor and consultant team, great learning environment, variety of patients” (Respondent 266, Female, AST)*
	*“Happy and friendly work environment. Appropriate expectations of registrar workload” (Respondent 204, Female, GPT3/PRT3)*
*Areas for improvement*	
Formal/ structured teaching	*“More direct supervision and formal teaching sessions would be required for a GPT1/2 registrar” (Respondent 12, Female, GPT2/PRT2)*
	*“Supervision. But this is just the nature of this practice. It’s busy and teaching isn’t the focus” (Respondent 37, Female, GPT2/PRT2)*
	*“There needs to be more variety and time dedicated to education hours” (Respondent 42, Male, GPT1/PRT1)*
	*“On site clinical supervision and teaching could be improved especially for GPT 1” (Respondent 224, Female, GPT1/PRT1)*

### Strengths of the educational environment

Two main themes emerged in relation to the registrars’ perception of the strengths of the educational environment – [1] supportive and accessible supervisors [2] good teamwork.

#### Supportive and accessible supervisors

The registrars’ indicated that a major strength of the educational environment was having “supportive and accessible supervisors”. The registrars commended the supervisors in their training posts for being supportive, approachable and for creating great learning environment - “*Very approachable and supportive supervisor who has excellent knowledge and approach to general practice and keen dedication to teaching*” -*(Respondent 333, Male, GPT1/PRT1*). They also stated that the working environment was extremely supportive for registrars with easily accessible and knowledgeable supervisors - “*Excellent support, good supervisor role modelling, and strong clinical knowledge of supervisor*” - *(Respondent 112, Male, GPT2/PRT2*).

#### Good teamwork

The registrars also indicated that the educational environment was friendly and encouraged teamwork - “*Teamwork, friendly and approachable”* - *(Respondent 206, Female, GPT1/PRT1)*; *“Good team work, good support, lots of doctors approachable and willing to help” - (Respondent 158, Male, Extended Skills PRRT4)*. The registrars also stated that the educational environment provided them with good learning opportunities. One respondent stated that the practice had *“Good teaching and supervision, with e*x*cellent mix of cases” - (Respondent 170, Male, GPT2/PRT2)*. Another respondent stated that the strength of the placement was *“Teaching and variety of clinical presentation” - (Respondent 108, Male, GPT2/PRT2)*.

The themes identified by the registrars as strengths of the educational environment are in congruence with the three highest rated items on the SPEED tool (Table 2) which relate to accessibility of the supervisors (The supervisors are respectful towards registrars = 4.52; The supervisors are approachable and helpful = 4.48; and I feel part of the team working here = 4.42).

### Areas for improvement

In terms of areas for improvement, the registrars requested for more formal teaching sessions - *“more direct supervision and formal teaching sessions would be required for a GPT1/2 registrar” - (Respondent 12, Female, GPT2/PRT2); “On site clinical supervision and teaching could be improved especially for GPT 1” (Respondent 224, Female, GPT1/PRT1); “Supervision. But this is just the nature of this practice. It’s busy and teaching isn’t the focus” (Respondent 37, Female, GPT2/PRT2).* The request for more formal /structured teaching could be linked to the observed lowest mean scores for the items “my supervisor prevents me from having to perform too many tasks irrelevant to my learning” = 3.92 and “my supervisor reserves time to supervise/counsel me” = 3.98.

## Discussion

This study aimed to validate and assess the reliability of the SPEED as well as explore the overall satisfaction and perceptions of registrars in relation to a newly developed postgraduate GP training program. The exploratory factor analysis and the principal component analysis negate the proposed underlying components for the original SPEED tool. Even though there are no studies reporting validity evidence of the SPEED tool based on its internal structure, our results echo the findings of Boor et al. [1] and Koutsogiannou et al. [28] who reported that only one component explained the variance in the 40 items of the Postgraduate Hospital Educational Environment Measure (PHEEM). This finding may be an indication that factors which influence the educational environment are numerous, complex and highly inter-related [29]. In addition, it is not surprising that the 15 items map to a single factor, because they are almost all concerned with quality of supervision. The authors of the original paper raised the possibility that without the other items from their original 41-item questionnaire, the performance may differ. This study could not replicate the factorial validity analysis of the tool performed by the original authors. Further evaluation of the SPEED tool in various settings is therefore required to validate the findings in this study.

Nevertheless, the results of our study showed that the SPEED tool has an excellent internal consistency in our setting with person reliability estimates ranging from 0.87 to 0.93, which are results similar to the value (0.9) reported by the original authors [14]. More importantly, this study provides multiple sources of validity evidence for the SPEED tool, from exploratory factor analysis and from the many-facet Rasch model. Principal component analysis is not a formal structural model per se, and it does not contain error terms, so it is useful to index the variables but it cannot be used to gather validity evidence. Given that the participants in the study are registrars in a rural generalist-training program with a distributed model, the results showed that the SPEED is a tool for assessing the educational climate in a rural medical postgraduate GP training context with a high degree of validity and reliability.

Overall, the registrars agreed that the educational environment is of high quality, with most (13 out of 15) of the items been rated above 4 out of 5. The higher scoring observed in this study may be as a result of the 5-point scale used in comparison to the 4-point scale used by the original authors. Evidence from the literature suggests that satisfaction with the educational environment is influenced by the perception of the educational environmental quality [30, 31]. This implies that the educational environment within JCU’s GP distributed training model is positive and of good quality. In addition, there were no subgroup differences in satisfaction rates in relation to age, training term or location of university training. This indicates that the SPEED can be applied to diverse settings. However, female registrars gave higher satisfaction ratings than their male counterparts on two SPEED items – “The practice/post staff are clear about my duties and responsibilities” and “My supervisor reserves time to supervise/counsel me”. This finding contrasts with a previous study which reported that male residents were more satisfied with the training environment compared to female residents [32], though the study used a different tool.

Highest mean scores were obtained in items that emphasised a supportive educational environment- “The Supervisors are approachable and helpful, and I feel part of the team working here”. Furthermore, strengths of the educational environment as identified by the registrars in the open-text responses were supportive and accessible supervisors, and good teamwork. These strengths underscore the findings from the SPEED tool in which accessibility of the supervisors and good teamwork were rated highly by the registrars. In addition, the perceived availability and accessibility of the supervisor and teamwork reflect the importance of interactions in the clinical educational environment. As reported by Cannon et al., medical trainees place higher values on the interactions in the learning environment than on teaching styles [8]. In relation to the human environment theory, teamwork and supervisor accessibility relate to the relationship domain and emphasise the value of interaction and collaboration with others in a conducive educational environment [33]. These interactions and collaborations are important for the learning process, effective teaching and for achieving learning goals [15].

The items with the lowest mean scores on the SPEED were “*My supervisor prevents me from having to perform too many tasks irrelevant to my learning*” and “*My supervisor reserves time to supervise/counsel me*”. Given that the clinical training/educational environment is a work setting, balancing service and clinical education may be challenging. According to Galvin and Buys, tasks with little or no educational value were considered by residents to potentially interfere with training [34]. These tasks are viewed negatively and may be considered irrelevant to clinical education [34]. In addition, having structured didactic sessions may be difficult with the clinical workload and possibly registrars who have just concluded internship may still want the dedicated formal teaching style that occurs in undergraduate medical education [35]. In addition, a mismatch between an instructor’s teaching style and the learner’s method of learning may lead to potential learning obstacles [36, 37]. Nevertheless, understanding the learner’s dominant learning style and using effective teaching strategies that match the learner’s need may motivate them and improve their perception of the educational environment [2, 38]. Adjusting learning styles while effectively incorporating the workload of the practice may help the registrars expand their ability to learn while working [38]. In this context, the words supervise, and counsel depicted guidance. However, we acknowledge that the words supervise, and counsel may have been misconstrued by the respondents, and this may have contributed to the low mean score.

### Implications for practice and future research

The SPEED is a concise and reliable tool that assesses the quality of postgraduate medical educational environment. However, in our setting, both exploratory factor analysis and principal component analysis failed to support the three factors suggested in the tool, as it could be suspected by the high reliability coefficients obtained in this study and the original study of the SPEED tool. We acknowledge that changing the Likert scale points from 4- points as used in the original study to 5-point in our study may alter response process and affect response process validity. Indeed, previous studies have shown that number of response options affects the psychometric properties of a scale [39, 40]. However, as the number of response alternatives increases, the reliability and validity of the scale are better [40]. This was reflected in our study with reliability estimates slightly higher than those reported by the original authors. However, we could not empirically replicate the factorial validity analysis of the tool. The findings of our study show that the tool is unidimensional with inter-related domains/items. According to the human environment theoretical framework, the relationship dimension assesses the extent to which individuals support and help each other and the extent to which they are involved with the environment [26]. This is reflected in this study which showed that support, interaction and collaborations of the supervisors with registrars resulted in a perception of a good learning environment. In addition, items which addressed components of teaching and learning reflect the personal development dimension of the human environment which assess the basic directions along which self-enhancement and personal development tend to occur [26]. Overall, the SPEED is a concise and valid tool which is applicable in a rural GP training setting. Future research might focus on validating the tool in other settings.

### Strengths and limitations

The main strength of this paper is the validation of the SPEED in a rural generalist medical training program. To the best of our knowledge, we are the first to validate the tool in a different clinical environment with a distributed training model. However, modification of the tool to suit our setting may have led to higher scoring and subsequently influenced the assessment of the tool’s validity. This study focused on validating the tool and assessing its appropriateness for our setting and not on measuring the educational environment in rural postgraduate medical training centres. Therefore, the results relating to registrars’ satisfaction with the educational environment should be interpreted with caution. Furthermore, our study may not be generalisable to other postgraduate specialist medical training settings and should therefore be interpreted with caution, particularly given the relatively small size of the two convenience subsamples used in this study. Further research is required to determine the timing and frequency of administration of the tool during residency training.

## Conclusions

In conclusion, the cross-validation process employed in this study confirms that the SPEED is a concise tool with only 15 items that can be used to measure the quality of the educational environment in postgraduate GP training with high degrees of validity and reliability. The SPEED is based on a theoretical framework that emphasises the importance of interactions in the clinical educational environment and fosters easy identification of areas for improvement. However, when applied in a new setting, the tool may not function as a multidimensional tool consistent with its theoretical grounding.

## Abbreviations

EFA: Exploratory factor analysis
GMT: Generalist Medical Training
GP: General Practice
JCU: James Cook University
MFRM: many-facet Rasch model analysis
PCA: Principal factor analysis
PGME: Post Graduate Medical Education PGME
RMSEA: Root mean square error of approximation
SPEED: Scan of Postgraduate Educational Environment Domain
TLI: Tucker–Lewis index
